# Keratin/chitosan film promotes wound healing in rats with combined radiation-wound injury

**DOI:** 10.1007/s10856-025-06860-z

**Published:** 2025-01-27

**Authors:** Yu-mei Wang, Tong Xin, Hao Deng, Jie Chen, Shen-lin Tang, Li-sheng Liu, Xiao-liang Chen

**Affiliations:** Department of Nuclear Medicine, Chongqing University Cancer Hospital, No. 181 HanYu St, Shapingba District, Chongqing, 400030 PR China

## Abstract

Human hair keratin, a natural protein derived from human hair, has emerged prominently in the field of wound repair, showcasing its unique regenerative capabilities and extensive application potential. However, it is a challenge for the keratin to efficiently therapy the impaired wound healing, such as combined radiation-wound injury. Here, we report a keratin/chitosan (KRT/CS) film for skin repair of chronic wounds in in rats with combined radiation-wound injury. In brief, the KRT/CS film was characterized by scanning electron microscopy (SEM), mechanical property analysis, water absorption, and swelling analysis. A rat model of combined radiation-wound injury was employed to evaluate the therapeutic efficacy of the KRT/CS film. Finally, the systemic biotoxicity of KRT/CS film was assessed through histological analysis. The surface of KRT/CS film was uniform and smooth compared with the KRT film, and the mechanical property, swelling rate and water absorption rate of KRT/CS film were significantly improved, which can meet the application requirements of wound excipient dressing. Furthermore, the combined radiation-wound injury in rats was established that the wound closure rate was achieved 74.46% after 14 days of treatment with KRT/CS film, comparing to the single KRT membrane and commercially available Band-Aids. Histological analysis demonstrated that the amount of angiogenesis and collagen deposition in wounds treated with KRT/CS were significantly improved. These findings demonstrate the KRT/CS film as a promising therapeutic agent for combined radiation-wound injury.

**Graphical Abstract:**

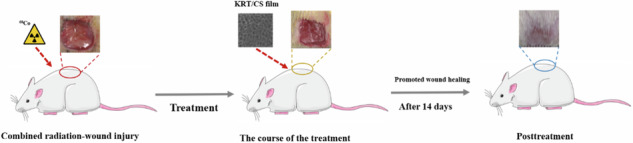

## Introduction

Radioactive substances are used widely in the medical and agriculture industries. In the medical field, these substances are commonly used for tumor diagnosis and treatment. Approximately 50% of cancer patients receive radiotherapy at some stage in the treatment process [1]. While radiation attacks tumor lesions, it also causes irreversible damage to normal cells, and a major side effect is damage to healthy tissues, particularly skin tissues [2]. In radiation therapy, the skin is the first organ exposed to ionizing radiation, which is not only often suffers from radiation injury but also particularly susceptible to compound injuries. With the continuous progression of the wound, local ulcers may ensue, leading to the radiation combined injury, and systemic infection, acute bleeding, and other complications may arise in severe cases. In wound healing, several cytokines and growth factors are produced via paracrine or autocrine modes [3], and the factors mediate and regulate the cascade response to promote wound healing [4]. However, Ionizing radiation and mechanical trauma can significantly impair the process of wound healing by inducing detrimental effects on the wound. These forms of injury disrupt the functionality and reduce the population of repair cells, diminish the availability of essential growth factors, hinder collagen synthesis, and dysregulate inflammatory cell responses, collectively compromising the overall efficiency of tissue repair and regeneration. Consequently, the healing progress of compound injury caused by radiation heals more gradually than radiation injury or usual wounds [5]. While numerous studies have focused on the response of individual tissues to radiotherapy injuries, relatively few investigations have specifically concentrated on tissues subject to non-radiation-induced compound injuries. In clinical practice, treatment strategies for combined radiation-wound injury encompass multiple dimensions, including pharmacological therapy, local wound management, and physical interventions, aiming to promote wound healing, alleviate symptoms, and improve patients’ quality of life. Clinically, bandages, hydrogel materials, and silver ion dressings are commonly used to manage wounds. However, each of these approaches has certain limitations. Bandages, for instance, tend to adhere to the wound surface, which can result in additional mechanical trauma during dressing changes. Hydrogel materials, although effective in hydrating the wound bed, possess a highwater content and are limited in their ability to absorb substantial amounts of exudate. Moreover, while silver ion dressings demonstrate rapid antibacterial efficacy, their fibers are prone to shedding, which may compromise their therapeutic benefits. Nevertheless, despite demonstrating certain efficacy under specific conditions, these therapeutic approaches face significant limitations and challenges, which remain critical issues requiring urgent resolution in current research.

Wond healing is a lengthy and complex regenerative process and the use of biomaterials is an effective method to improve wound healing by mediating the establishment of an immune microenvironment that promotes the adhesion and proliferation of relevant skin repair cells. Since the early 1980s, various biological dressing materials have been developed for the promotion of wound healing [6]. Among these, keratin (KRT)-based biomaterials have demonstrated excellent properties in accelerating the healing process. KRT, as an ideal biomaterial, has various favorable properties, such as rapid hemostasis, promotion of wound healing, and peripheral nerve repair [7–11]. Moreover, it was shown to accelerate wound healing in both animal and clinical trials, and KRT-based wound repair products have been promoted in both domestic and international markets. For example, KRT-based products were administered to 22 patients with prolonged venous leg ulcers with conventional compression dressings. The results showed a wound healing rate of 71% after 24 weeks of treatment with KRT [9]. However, poor mechanical properties are a major drawback of KRT products, which severely affects their clinical application. Therefore, multiple studies have been conducted to improve the mechanical properties of these products by cross-linking them with other materials [12–14]. This approach not only allows the application of KRT to a wider range of uses but also helps achieve a synergistic healing effect.

Chitosan is a cationic polysaccharide derived from chitin. It is considered to be the second most abundant polysaccharide after cellulose [15, 16]. As a natural biomaterial that has been widely studied and applied in multiple biomedical applications, CS is an important candidate for the development of film-based products not only owing to its good biocompatibility, biodegradability, and antimicrobial properties but also owing to its good mechanical stability and film-forming ability [17–21]. Currently, CS is widely used in many fields, such as in the development of tissue engineering materials, medical fibers, antimicrobial agents, and slow drug-releasing materials, as well as in chemical industries [22, 23].

Based on this, we innovatively developed a KRT/CS film as an advanced skin dressing in this study. We systematically investigated its mechanical properties, swelling behavior, in vivo degradation characteristics, and its potential application in the healing of radiation-induced complex wounds. By introducing chitosan, the mechanical weakness of KRT when used alone was effectively compensated, significantly enhancing the overall structural strength of the composite film. Owing to its excellent biocompatibility and controllable biodegradability, the prepared dressing can not only gradually degrade in accordance with the wound healing process but also significantly reduce the frequency of dressing changes, thereby minimizing patient pain, decreasing potential damage to the new epidermis, and effectively mitigating the risk of infection. In summary, this innovative KRT/CS composite film, with its unique structural and performance advantages, demonstrates significant application advantages and broad prospects in the field of radiation-induced complex wound dressings. It can not only effectively promote wound healing but also provide a comfortable healing environment, alleviate patient suffering, and offer a new and effective solution for the treatment of radiation-induced skin injuries.

## Materials and methods

Low deacetylated chitosan (CS, Shanghai Macklin Biochemical Technology Co., LTD.); Keratin (KRT, 40–66 kDa Laboratory extraction); Acetic acid (Aladdin Holdings Group Limited); Band-Aid (BA, Guangzhou Tuoneng Pharmaceutical Co. LTD); 10% Chloral Hydrate (w/v, Shanghai Macklin Biochemical Technology Co., LTD.); Sodium Dodecyl Sulfate (SDS, Sigma-Aldrich Corporation of America); Trimethylaminomethane (Tris, Nanjing Senbeijia Biotechnology Co., LTD.); Thioglycolic acid (TGA, Chengdu KELon Chemicals Co., LTD) and 4% Paraformaldehyde (w/v, Shanghai Macklin Biochemical Technology Co., LTD.); Penicillin injection (Harbin Pharmaceutical Group Pharmaceutical General Factory); HE Staining Kit (Regen Biotechnology Co., LTD.); Masson Dye Kit (Regen Biotechnology Co., LTD.); Tris-HCl Buffer Solution (Nanjing Senbeijia Biotechnology Co., LTD.); Xylene (Chongqing Chuandong Chemical Co., LTD.); HaCat cells (Zeye Biotechnology Co., LTD, Shanghai); DMEM medium, fetal bovine serum, trypsin, penicillin-streptomycin solution (100×), and dimethyl sulfoxide (DMSO) were procured from Sigma； CCK8 kit, RNAiso plus.

### The extraction of human hair keratin

Keratin was extracted from human hair according to a method described by a previous study [24]. Firstly, human hair (50 g) was accurately weighed, and subsequently, TGA (57.72 mL) was dissolved in water (1500 mL) to prepare a 0.5 M TGA solution. Finally, the pH of the solution was then adjusted to 11. Human hair was washed using 0.5% SDS, and treated with TGA for 15 h. The reduction solution was collected and the crude fraction was extracted first with Tris base (100 mM) and then with deionized water. The pH of the extracted solution was adjusted to 4.0, and the mixture was centrifuged at 6000 rpm for 40 min at 4 °C.

### Preparation of KRT and KRT/CS composite membranes

KRT and KRT/CS composite membranes were prepared according to a method reported by Tanabe et al. [25], with minor modifications. At room temperature, 25 mg of chitosan was stirred in 10 mL of 75% acetic acid until a uniform solution was obtained. Three times the volume of glacial acetic was added to an aqueous solution containing 10 mg of KRT. After the KRT was completely dissolved, it was mixed with an appropriate volume of chitosan solution. The KRT solution and KRT/chitosan were poured into a glass Petri dish with a diameter of 5 cm × 5 cm at the bottom and dried overnight at 50 °C. The average thickness of the films was 0.01–0.02 mm. All composite membranes were irradiated with UV light for 15 min prior to their utilization.

### Surface morphology analysis of KRT and KRT/CS films

SEM was used to analyze the surface morphology of the materials. Because of advantages such as high resolution and a large field of view, SEM is used to observe the fine structure of the surface of composite films. The composite film was dried overnight at room temperature, and materials of appropriate sizes were cut and placed on conductive glue for reserve. The surface morphology of the KRT and KRT/CS composite films was observed at an accelerated voltage of 5.0 kV. Before imaging, the materials were sputtered with gold for 20 s and then subjected to measurement.

### Dilatability and water absorption tests of KRT and KRT/CS composite membranes

The KRT and KRT/CS composite films were cut and allowed to expand into squares of 2 cm × 2 cm. These were immersed in Tris-HCl buffer solution (pH 8.9) at room temperature till they were of a consistent size. The equilibrium swelling ratio and water absorption were calculated according to Eqs. (1.1) and (1.2):

1.1 $${{Expansion}}\,{{Rate}}( \% )=\frac{S{{wet}}-S{{dry}}}{S{{dry}}}\times 100$$

1.2 $${{Swelling}}\,{{Rate}}( \% )=\frac{Mt-M0}{M0}\times 100$$

Where *S*dry and *S*wet are the areas of the dry and swollen films, respectively. *Mt* is the weight at any time point, and *M0* is the initial weight.

### Assessment of the mechanical properties of KRT and KRT/CS films

After drying, the film was cut and allowed to expand to 2 cm and 1 cm in width. The thickness “*d*” was 0.01 mm, as measured using a micrometer. The tensile test instrument was used for testing. The stretching speed was set at 10 mm/min, and the test was repeated three times to calculate the average value according to the following formula:

1.3 $$Y=\frac{\delta }{\varepsilon }$$

1.4 $$\varepsilon =\frac{L-L0}{L0}$$

1.5 $$\delta =\frac{P}{b\,\ast \,d}$$

Where *Y* is Young’s modulus, *δ* is fracture stress, *ε* is elongation at break, *P* is tensile strength, and *L* is tensile length.

### Establishment of Sprague-Dawley (SD) rats models with radiation-wound injury and skin trauma

A full-thickness wound model was established to assess the repair performance of KRT and KRT/CS composite membranes in radiation-wound injury. The study was approved by Ethics committee of Chongqing University Cancer Hospital, China. Animal experiments were conducted in accordance with the guidelines of the institutional Animal Care and Use Committee of Chongqing University Cancer Hospital, China. According to the modeling method used, SD rats were randomly divide into five groups (*n* = 3), namely the wound injury, radiation-wound injury, band-aid (BA), KRT, and KRT/CS groups. Male SD rats (weight: approximately 180 g) aged 4–8 weeks were housed in a constant temperature chamber (at 22–25 °C, under a light/dark cycle) with access to food and water during the study period. The rats (except those in the normal wound group) were depilated on the dorsal surface 1 day before irradiation, gas-anesthetized with 10% Chloral Hydrate, and irradiated with γ-rays from a cobalt source (^60^Co) at an absorption rate of 5.0–5.7 mGy/s (duration: 1000 s) and a total dose of 5 Gy [5, 26]. The dorsal surfaces of SD rats were sterilized under aseptic conditions using iodophor. A square of 1.0 cm × 1.0 cm was marked on the skin with a marker pen, and an incision of 1.0 cm × 1.0 cm was made using surgical scissors according to the markings. Following this, the incised area was sterilized using iodophor. Sterilized KRT/CS composite membranes, KRT membranes, and BA were applied to the wounds, and the untreated wounds were used in the negative control group to record and observe the healing of the skin wounds. Images of the wounds were acquired at predetermined postoperative intervals (7th and 14th days), and the sizes of the wounds during the healing process were measured using ImageJ. The rate of wound repair was calculated according to the following formula:

1.6 $${{Wound}}\,{{healing}}\,{{rate}}( \% \,)=\frac{At-A0}{A0}\times 100$$

where *A0* is the initial area of the trauma site and *At* is the area of the trauma site at fixed time points (days 0,7, and 14).

### Hematoxylin-eosin (HE) and Masson’s trichrome (MTC)staining

Skin tissues were taken from the central section of the wounds on the 3rd, 7th, and 14th postoperative days, fixed using 4% paraformaldehyde (PFA), and stored in a refrigerator at 4 °C for use at a later instance. The skin tissue was dehydrated using a gradient with different concentrations of ethanol solution. After treatment with xylene to induce transparency, the skin tissues were embedded in paraffin and stained with HE and MTC before sealing. The tissues were then observed under a light microscope.

### In vivo degradation test in SD rats

The in vivo degradation of each group of protein-based biomaterials was examined by spraying them onto slides and implanting them under the skin of SD rats after drying naturally and sterilizing with ultraviolet light. In the BA group, the same shape and size of the protein-based materials was maintained. Male rats weighing approximately 180 g were divided into five groups (*n* = 3), namely Irradiated (IR), control, BA, KRT membrane, and KRT/CS groups. The rats were anesthetized with the same dose of 10% Chloral Hydrate (as described in subsection “Establishment of Sprague-Dawley (SD) rats models with radiation-wound injury and skin trauma”), their dorsal surfaces were shaved and sterilized with iodine vapor, and then they were fixed on the surgical table. Following this, the dorsal surfaces of the rats were incised under aseptic conditions, and the materials in each group were implanted in the 5 mm point of the incision. The rats were fixed on the operating table, and the dorsal surfaces of the rats were incised under aseptic conditions. The materials in each group were implanted at the site of incision at a distance of 5 mm, and the incision was sutured with sterile sutures. The wounds were sterilized with iodine vapor after suturing. The rats were housed in separate cages under aseptic conditions, and their survival was observed and recorded every day. Photographs were taken on the 7th and 14th postoperative days to observe the degradation of the materials in vivo. The rats were sacrificed on the 14th day, and their heart, liver, spleen, lungs, and kidneys were removed and fixed in 4% PFA for histological analysis.

### Cell viability assay

The cytotoxicity of the materials could be verified with cell proliferation assay. There are 5 groups, namely Control, IR, BA, KRT and KRT/CS (*n* = 3). Following cell adhesion, the cells were incubated with different materials for 24 h, 48 h and 72 h, respectively. For the Control and IR groups, PBS was added as a negative control. At the end of incubation, 10 μL of CCK-8 reagent was added to each well, followed by co-cultured with the cells in an incubator for 2 h. Subsequently, the absorbance at 450 nm was measured using a Microplate Reader (3550, Bio-Rad, USA). The cell viability in the control group was set to 100%, serving as the baseline for calculating the relative cell proliferation rates in the experimental groups.

### Statistical analysis

All experiments in this study were performed at least in triplicate unless stated otherwise. Data were presented as mean ± standard deviation (SD). One-way analysis of variance (ANOVA) followed by a post hoc Tukey’s multiple comparison test was used. The statistical significance level (*p*) was set at <0.05.

## Results and discussion

### Characterization of KRT and KRT/CS composite membranes

**Fig. 1:**
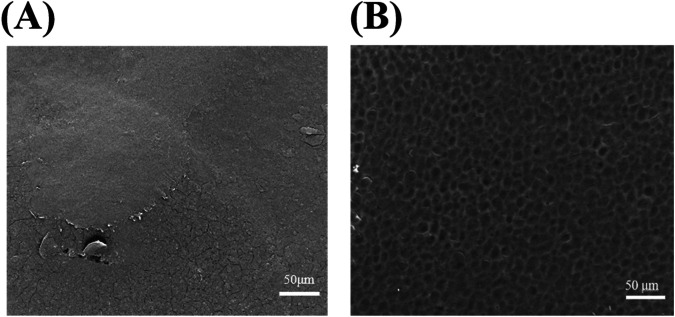
**A** The membrane surface of KRT, **B** SEM image of KRT/CS composite membrane surface

The surface morphology of pure KRT and KRT/CS composite membranes were analyzed with SEM. Figure 1A shows the scanning electron micrograph of the KRT membrane, and Fig. 1B shows the scanning electron micrograph of the surface of the KRT/CS composite membrane. The pure KRT membrane appeared a smooth, continuous, and uniform morphology without bead-like structures, although minor cracks were observed. However, subtle cracks appeared on the surface of the KRT film, which may be caused by the roughness of the keratin. In comparison, the surface morphology of KRT/CS composite membrane exhibited more distinctive features. While maintaining smooth and uniform multi-microporous characteristics and it is evident that the roughness increases in the presence of keratin and providing a more favorable microenvironment for cell attachment and growth [27]. Interestingly, despite being a composite of keratin and chitosan, no undissolved chitosan particles or aggregated keratin clusters were found in the SEM images. This strongly demonstrates the excellent compatibility and dispersion of the two components during the composite formation process. On closer inspection, the KRT/CS composite film surface also displayed microcracks, which align with previous studies [28, 29]. Moreover, Uneven distribution could lead to localized functional differences, ultimately impacting overall performance. The results suggest that smooth, uniform, and continuous KRT/CS composite membranes can be obtained using this method.

### Solubility analysis of KRT and KRT/CS films

**Fig. 2:**
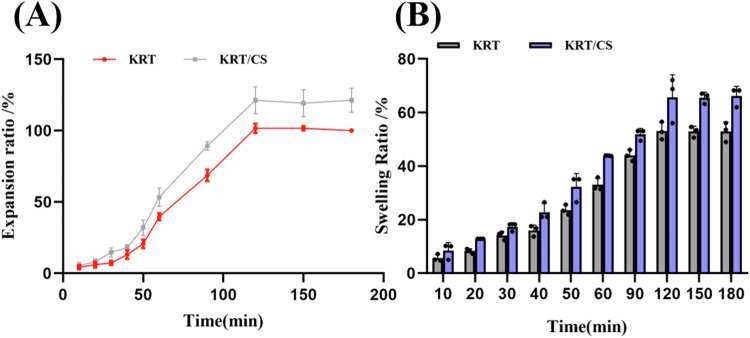
**A** Swelling rate of KRT/CS, **B** water absorption rate of KRT/CS

To verify the water absorption capacity of KRT and KRT-chitosan composite membranes, we investigated the swelling rates and water absorption rates of KRT and KRT/CS composite membranes at different time points and saturation was reached at 120 min. As shown in Fig. 2A, B, the swelling rate of the KRT membrane immersed in Tris-HCl buffer was 101.67% after 120 min, and the water absorption rate was 53.09%, highlighting the strong hydrophilic nature. In contrast, the KRT/CS composite membrane demonstrated even better swelling performance under the same conditions and the dissolution rate as high as 121%, and its water absorption rate was 65.63%. This improvement could be attributed to the complementary properties of two products. Chitosan, a natural polysaccharide, contains abundant hydrophilic groups such as hydroxyl and amino groups, while keratin also possesses intrinsic hydrophilic characteristics. Therefore, the synergy between two components significantly enhances the film’s capacity to absorb and retain water absorption. The water absorption is an important index for evaluating the application of biomaterials in the field of wound dressings and materials with better water absorption can prevent the accumulation of exudates in the wound and facilitate the absorption of nutrients into the dressing. In addition, the KRT/CS film, which has the excellent water absorption properties can rapidly absorbs the water present form wounds, maintaining a dry surface that supports faster recovery. Overall, the KRT/CS composite film demonstrates outstanding potential as a wound dressing material, providing enhanced water absorption performance that supports wound healing efficiently.

### Analysis of the mechanical properties of KRT and KRT/CS composite membranes

**Fig. 3:**
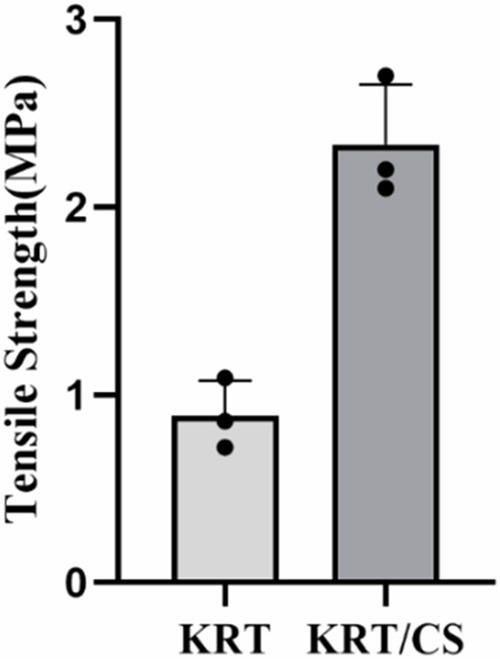
Tensile strength of KRT and KRT/CS films

An important property index of all materials has a direct and profound impact on the application scope and practical utility by mechanical properties. As shown in Fig. 3, the mechanical property of the KRT membrane was 0.89 Mpa, revealing mechanical limitations in the absence of any modification. In the absence of any additives, KRT is too fragile to handle and making it unable to withstand mechanical stress during processing, which severely limits their potential for broader applications. To address this mechanical shortcoming of KRT films, chitosan was introduced as an effective reinforcing agent. The results indicate that the strength and hardness of KRT increase upon the addition of chitosan. The mechanical property of the KRT/CS composite film was 2.5 Mpa in Fig. 3, which is close to the Young’s modulus of human skin (0.5–2 Mpa) [30]. Therefore, by adding chitosan to KRT membranes, we can prepare skin excipients with better mechanical strength that can withstand the wear and tear of daily life. This improvement in durability undoubtedly broadens the application potential of the KRT/CS composite film, making it a promising material for skin-related applications. Most importantly, given its excellent mechanical properties and biocompatibility, KRT/CS composite membranes also show potential applications in tissue engineering and drug delivery, among other uses. Therefore, it can be anticipated that the KRT/CS film will emerge as a multifunctional, high-performance biomaterial, injecting new vitality into the development of the biomedical field.

### Evaluation of in vivo radiation-wound injury healing

**Fig. 4:**
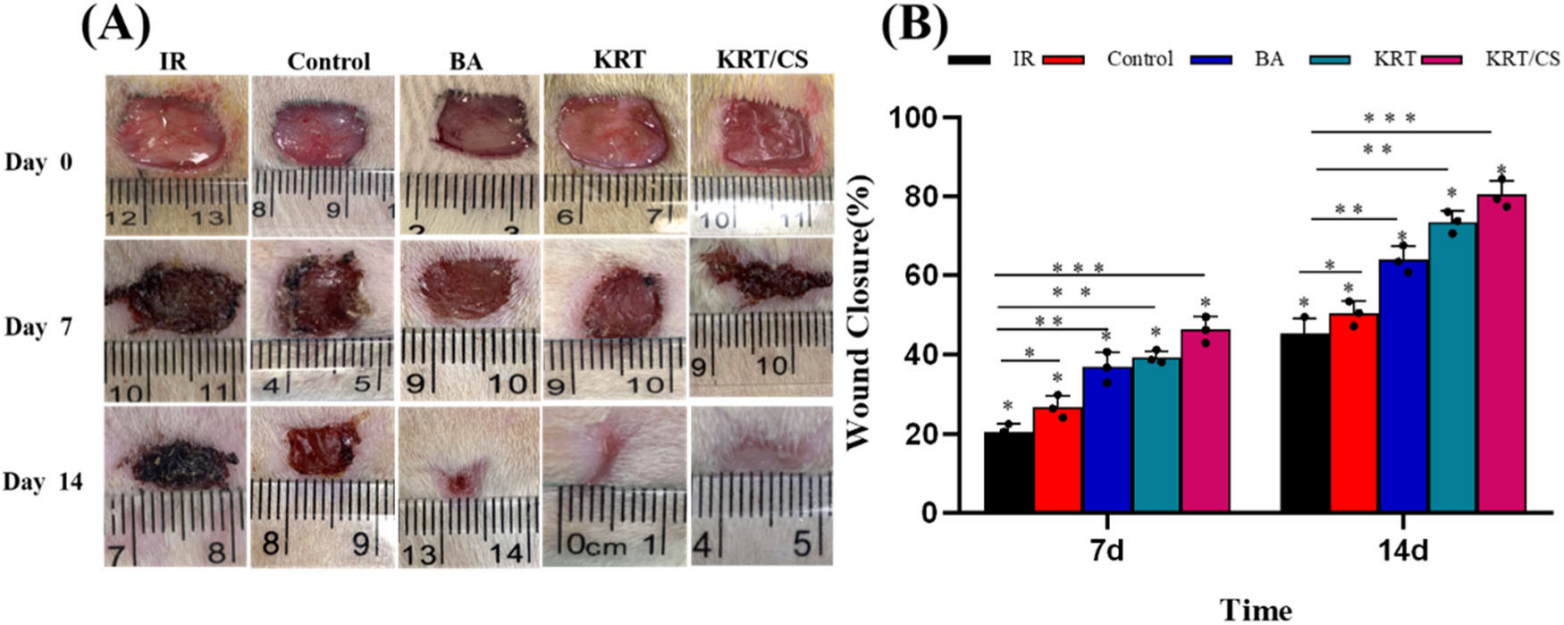
The effect of different materials on wound healing. **A** Wound healing in different time periods; **B** statistical graph of wound healing rate in different time periods ***p ≤ 0.001, **p ≤ 0.01 and *p ≤ 0.05

The radiation combined injury wound healing efficiencies of KRT and KRT/CS composite membranes were investigated in vivo in a full-thickness skin defect model, and the wounds were imaged at different time points after the KRT and KRT/CS composite membrane treatments. As shown in Fig. 4A, a scab was formed in all groups on day 7 after the trauma, but the rate of wound repair was different. The scab area was larger in the IR and control groups, whereas that in the KRT and KRT/CS groups was relatively smaller. On the 14th day, even though the area of the scabs in the IR and control groups was smaller than that on the 7th day, the scabs had not been removed completely. However, the scabs were completely removed in response to treatment with KRT, KRT/CS, and BA. The wound healing effect in the KRT/CS group was better than that in the other groups, and the effect in the KRT treatment group was comparable to that in the BA group. As observed in Fig. 4B, the degree of wound healing in the different groups decreased with time, with the KRT/CS composite film exhibiting greater healing than the KRT and BA films. A 34.51%, 36.83% and 42.61% reduction in wound size was observed in the BA, KRT, and KRT/CS groups on the 7th day after treatment, respectively. Meanwhile, in the IR and control groups, the wound size decreased only by 20.46 and 26.78%. On day 14 after treatment in the BA, KRT, and KRT/CS groups, the wounds were almost completely healed, with reductions of 63.31%, 63.97%, and 74.46% in the wound area, respectively, whereas a reduction of only 42.57% was observed in the IR group, and a reduction of 47.25% was observed in the control group. These results suggest that treating radiation combined injury is considerably more challenging than treating single radiation injury or mechanical injury healing; in such cases, KRT, KRT/CS, and BA can accelerate wound healing in 2 weeks. KRT/CS showed stronger potential for skin wound healing [31, 32]. After CS was introduced, the skin wound healing rate increased significantly compared with that in other groups, thereby opening up new avenues and approaches for the treatment of combined radiation injuries.

### Histological analysis

**Fig. 5:**
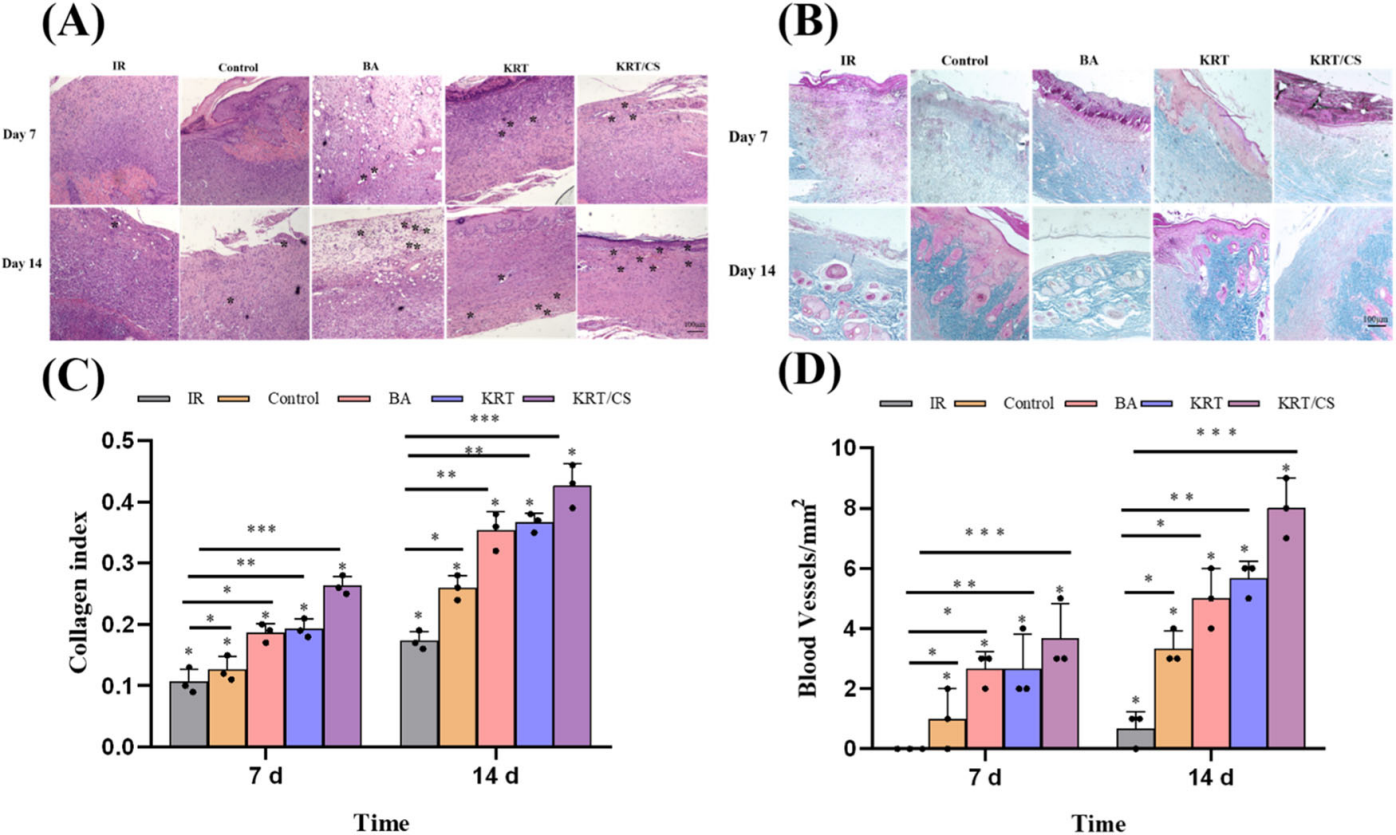
Tissue staining chart of each group at different time. **A** H&E staining diagram (*: Blood vessels); **B** Masson staining diagram; **C** number of angiogenesis; **D** collagen deposition ***p ≤ 0.001, **p ≤ 0.01 and *p ≤ 0.05

H&E and MTC staining are commonly used to characterize wound healing progress and evaluated via the histological examination. The degree of wound healing was primarily evaluated by quantifying the number of angiogenesis events (Fig. 5D), and collagen deposition (Fig. 5C) using direct counting methods. The H&E staining plots of each group at different time points (Fig. 5A). The results indicate that more neovascularization (black asterisk) was observed, with more neovascularization observed in the KRT/CS group but almost none observed in the IR and control groups. On day 14, neovascularization was observed in the IR and control groups, whereas neovascularization gradually increased in the KRT, KRT/CS, and BA groups, and the blood vessels were filled with red blood cells. However, the tissue arrangement could still not match the complete and regular arrangement observed in normal skin.

Collagen is the principal component of the skin and can restore the structural integrity of damaged tissue in wound healing progress [33]. Our findings showed that collagen plays an important role in the proliferative phase of wound healing by inducing granulation tissue formation and maintaining skin structure and elasticity. The wound healing effect of the materials was evaluated using the total collagen level [34]. Collagen deposition during wound healing was investigated by MTC staining (Fig. 5B). Compared with that in the IR and control groups, the epidermis was reconstructed more rapidly in the KRT, KRT/CS, and BA groups (on days 7 and 14 after the injury), and the level of newly formed collagen deposits under the epidermis increased after repair. The level of newly formed collagen deposits in the KRT/CS group was higher than those in the other groups, and the maturity was much higher than that in the other groups. These results indicate that KRT/CS has better wound repair ability than BA and can accelerate wound healing by promoting collagen formation.

### Biocompatibility test

**Fig. 6:**
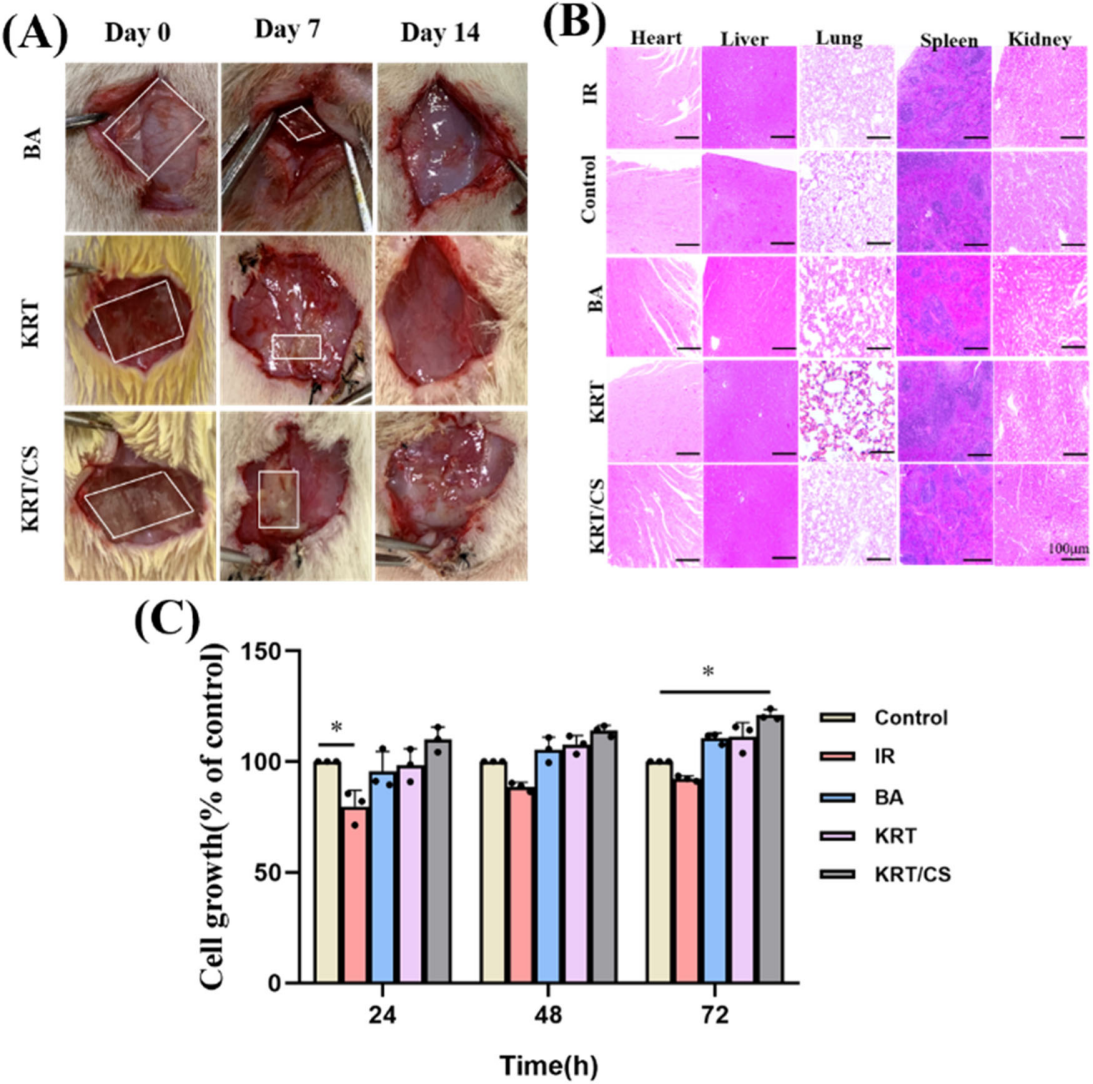
**A** Degradation of KRT and KRT/CS after implanted subcutaneously in vivo. **B** HE staining of main organs of rats on day 14 after subcutaneous implantation of different materials. (Scale bar = 100 μm). **C** CCK8 sasay for cell viability analysis after different incubation times

The in vivo biocompatibility of KRT, KKRT/CS, and BA was assessed in SD rats through subcutaneous implantation. None of the animals died at the end of the experiment, and no abnormal manifestations, such as wound infection and difficulty in movement, were observed. In addition, the biological safety in vitro of different materials was assessed and verified with CCK-8 assay. The in vivo degradation of the material at different time points in each group was observed based on a decrease in material size over time in Fig. 6A, and no erythema or edema was observed at the wound site [35]. However, according to the experimental data analysis on the 7th day, significant differences in degradation rates were observed among BA, KRT, and KRT/CS composite membranes. Notably, the degradation rate of the KRT/CS composite membrane was significantly slower than the other two. This finding indicates that the incorporation of chitosan effectively prolongs the duration of action of the composite membrane on the wound surface, thereby enhancing its repair efficacy. The data from the 14th day indicate that all materials have been degraded, indicating that the biocompatibility of the materials each group was good. The addition of CS did not affect the degradation rate of KRT in vivo. Figure 6B represents a plot showing the H&E staining patterns in each major organ on day 14, which was used to evaluate the degradability and toxicity of the implanted material. Compared with that in the IR and control groups, no obvious pathological tissue was detected in the BA, KRT, and KRT/CS groups after the implantation of protein materials in each group. No local failure was observed in cardiomyocytes, no inflammatory cell accumulation was observed in hepatocytes, and no pathological changes were observed in renal congestion and in other tissues [36]. CCK8 sasay for cell viability analysis after different incubation times in Fig. 6C, the BA, KRT and KRT/CS were added all exerted a proliferative effect on cells after 72 h, enhancing proliferation rates by 110.5%, 111.2%, and 121.1%, respectively. These results indicate that the two groups of KRT-based materials are highly biocompatible with good biosafety and can be used for radiation combined wound healing applications.

## Conclusion

Human hair KRT and chitosan are ideal skin dressing materials because of their good biocompatibility, high degradation rate, stability, and the ability to form secure structures. These have been used widely in medical dressings, especially in skin wound repair. Based on the characteristics of KRT, such as hemostasis and wound repair, KRT and CS were mixed to prepare a KRT/CS composite membrane with strong mechanical strength that was easy to use, had a short onset time, and showed hemostatic and healing properties. This material was used for repairing radiation-induced skin wounds in SD rats. After KRT/CS treatment for 14 days, the wound healing rate increased significantly compared with that in other groups; it was 74.46% in the KRT/CS group, whereas it was only 42.57% in the IR group. The results show that, compared to KRT, the KRT/CS composite membrane showed a stronger ability for repairing radiation combined injury tissue repair. We also observed that radiation combined injury is more challenging to treat than single radiation injury or mechanical injury. Biopsy of the wound site at 7 and 14 days revealed that treatment with KRT/CS composite membrane resulted in increased angiogenesis, and collagen deposition, indicating a favorable treatment outcome. Collectively, the portable KRT/CS composite membrane, with its remarkable ability to accelerate cell migration, proliferation, and angiogenesis, has emerged as an ideal biocompatible wound dressing, exhibiting significant efficacy in promoting the healing of radiation-induced complex injuries. Its unique structural and performance advantages position it as a highly promising candidate for applications within the biomedical field. As we look to the future, with the continuous deepening of research on KRT/CS composite membranes and the further advancement of technology, we are confident that this innovative dressing will play an increasingly pivotal role in the treatment of radiation-induced skin injuries, ultimately bringing relief and benefits to a greater number of patients and driving substantial progress and development in medical technology.

## Data Availability

The datasets used and/or analyzed during the current study available from the corresponding author on reasonable request.
